# Gray Model Study of Strength and Pore Structure of Recycled Concrete Powder (RCP) Concrete Based on Low-Field NMR Technology

**DOI:** 10.3390/ma16176058

**Published:** 2023-09-04

**Authors:** Yongli Hou, Zhengxing Yu, Jianhua Zhang, Hongrui Yang, Weiqing Song

**Affiliations:** 1 College of Civil Engineering, Inner Mongolia University of Technology, Hohhot 010051, China; houyongli@imut.edu.cn (Y.H.); 20201100302@imut.edu.cn (Z.Y.); 20211100326@imut.edu.cn (H.Y.); 20211100318@imut.edu.cn (W.S.); 2 Inner Mongolia Autonomous Region Engineering Research Center of Structure Inspection, Appraisal and Safety Assessment, Inner Mongolia University of Technology, Hohhot 010051, China; 3 College of Aerospace and Civil Engineering, Harbin Engineering University, Harbin 150001, China

**Keywords:** RCP, mechanical property, pore structure, gray entropy correlation grade, GM (1, 4)

## Abstract

In order to improve the resource utilization of recycled concrete powder (RCP), this study aimed to investigate the effect of RCP admixture, curing age, and alkali excitation on the strength of RCP concrete. In addition, the pore structure characteristics of RCP concrete were analyzed in combination with low-field NMR. Furthermore, a gray predictive GM (1, 4) model was established to predict the mechanical properties of the concrete based on the pore structure parameters, especially the compressive and flexural tensile strengths. The results of the study indicate that the mechanical properties, namely compressive strength and flexural strength, of RCP concrete exhibit an initial increase followed by a subsequent decrease with increasing RCP content at 3 d, 7 d, and 28 d curing ages. In particular, the concrete exhibits the highest mechanical properties when the RCP content reaches 10%. As the curing age increases, the RCP gradually achieves full hydration, resulting in further refinement of the concrete pores and a denser structure, which subsequently improves the mechanical properties. In addition, the strength growth rate of alkali-excited recycled concrete (ARC) showed a continuous increase, indicating that alkali excitation increasingly improved the mechanical properties of the concrete. Furthermore, the study accurately predicted the mechanical properties of RCP concrete by using GM (1, 4) prediction models for its compressive strength and flexural tensile strength using pore characteristic parameters.

## 1. Introduction

Since the beginning of the 21st century, there has been rapid global urbanization, resulting in significant amounts of construction and demolition (C&D) waste [1,2,3]. For example, the United States and the European Union generate approximately 700 million tons and 850 million tons of C&D waste annually, respectively [4]. In addition, China’s annual C&D waste generation has exceeded 3 billion tons [5]. Unfortunately, the current C&D waste utilization rate in China is only 30%, which is much lower than that of developed countries such as Europe and the United States (90%) and Japan and Korea (95%) [6]. The contradiction between the annual increase of C&D waste and the increasing scarcity of construction materials will seriously hinder the development of urbanization. According to the China Building Energy Consumption and Carbon Emission Research Report (2022) [7], the total carbon emissions from the entire construction process in 2020 amounted to 5.08 billion tons, accounting for 50.9% of the country’s total carbon emissions. As a result, carbon reduction in the construction sector has become an important aspect of China’s efforts to achieve carbon peak and carbon neutrality. The comprehensive promotion of C&D waste recycling is one of the key strategies to achieve these goals.

The processing of C&D waste can result in the production of recycled aggregate through a series of treatments. Although the performance of recycled aggregates may not be equal to that of natural aggregates, by improving the crushing process, surface treatment, and matrix modification, as well as adjusting the matching ratio, recycled aggregate concrete or mortar can still meet the design requirements. This has been demonstrated in previous studies [8,9,10,11,12]. However, the application of recycled concrete powder (RCP), which accounts for approximately 10–20% of the total mass produced during the preparation of recycled aggregates and has a particle size smaller than 0.075 mm, is still in the experimental research stage. It has been found [13,14,15,16] that RCP contains significant amounts of SiO_2_, Al_2_O_3_, and CaO and exhibits properties typical of supplementary cementitious materials (SCM), such as the micro-aggregate filling effect and the volcanic ash effect. Ma [17] has shown that RCP has a certain hydration activity and the ability to improve the pore structure. As a result, it can be used as an SCM to replace part of the cement, thereby promoting cement hydration and improving the properties of concrete materials. The mechanical properties of RCP concrete are highly dependent on the amount of RCP added. At present, some scholars [18,19] argue that the incorporation of a suitable amount of RCP can effectively improve the mechanical properties of concrete. Initially, increasing the amount of RCP in the mix leads to an improvement in the mechanical properties, but this improvement reaches a plateau and eventually declines. Yu [20] shows that the cubic compressive strength and tensile splitting strength of recycled aggregate powder concrete (RAPC) increase and then decrease with the increasing RCP substitution rate. The maximum values are obtained at a substitution rate of 15%. However, the research results of some scholars [21,22,23,24] are different from the above, and it is believed that the incorporation of RCP to replace cement will reduce the mechanical properties of concrete. Xiao [25] shows that the addition of RCP significantly affects the mechanical properties of sulfate aluminate cement-based materials. As the RCP content increases, the calcium alumina and aluminum hydroxide contents decrease, while the SiO_2_ content increases. Consequently, the compressive strength decreases as the RCP content increases.

Concrete is a complex and heterogeneous multiphase material that forms numerous pores during the setting and hardening process. These pores have different morphologies, complex structures, and a wide range of sizes. At present, there is limited research on the pore structure of concrete after the addition of RCP. Most scholars [26,27] have mainly relied on the mercury-in-pressure (MIP) method for analysis. Liang [28] observed that the incorporation of 6% RCP refined the pore structure of the slurry, resulting in a decrease in total porosity to 8.04% and a significant reduction in the proportion of large capillaries over 200 nm. This resulted in an improvement in compressive strength. However, there are few studies that investigate the microscopic improvement mechanism of concrete through the analysis of RCP using low-field nuclear magnetic resonance (NMR). Low-field NMR technology, as a non-destructive means of testing, has the characteristics and advantages of being non-destructive and can be retested many times, and has been widely used in medicine, agriculture, energy, geotechnical engineering, and research related to concrete pore structure. Therefore, more research is needed in this area. The relationship between the pore structure of concrete and its strength has been studied extensively, with much attention paid to establishing quantitative relationships between pore structure parameters and mechanical properties [29,30,31]. For example, Li [32] conducted compressive strength and pore structure tests on plain concrete and iron tailing powder concrete, and established a quadratic function that relates the fractal dimension of the pore surface, the specific surface area, the sensitive pore size range, and the compressive strength. Zhang [33] analyzed the pore structure characteristics of self-compacting concrete (SCC) with different aggregates using the low-field NMR method. They further investigated the effect of pore structure on the compressive strength of SCC through the theory of gray correlation entropy. In addition, a GM (1, 3) prediction model for concrete compressive strength was established. The above studies contribute to our understanding of the relationship between pore structure and concrete strength.

At present, there is relatively little research on the relationship between pore structure parameters and mechanical properties after the incorporation of RCP concrete. In addition, the investigation of the laws and quantitative relationship between pore structure and mechanical properties of RCP concrete under the influence of alkali excitation is particularly lacking. Therefore, this research focuses on the use of RCP derived from C&D waste in the resource recovery process. It aims to investigate the effect of replacement rate and curing age of RCP as well as alkali excitation on the mechanical properties of concrete. In addition, low-field NMR analysis is used to investigate the pore structure characteristics of the concrete. A GM (1, 4) gray prediction model is then established to correlate the mechanical properties of the concrete with the pore structure parameters. The primary objective of this study is to increase the resource utilization rate of RCP while reducing the environmental pollution associated with traditional cement production. This research has strategic significance for China’s building materials’ industry to achieve the “double carbon” goal [34] and effectively utilize bulk solid waste resources.

## 2. Experimental

### 2.1. Raw Materials

#### 2.1.1. Cementitious Materials

**Figure 1:**
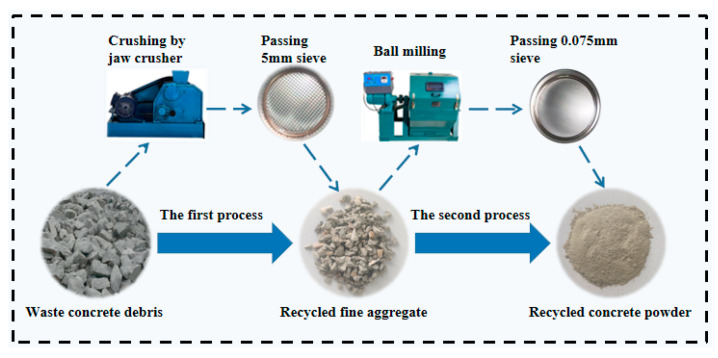
RCP preparation process.

**Figure 2:**
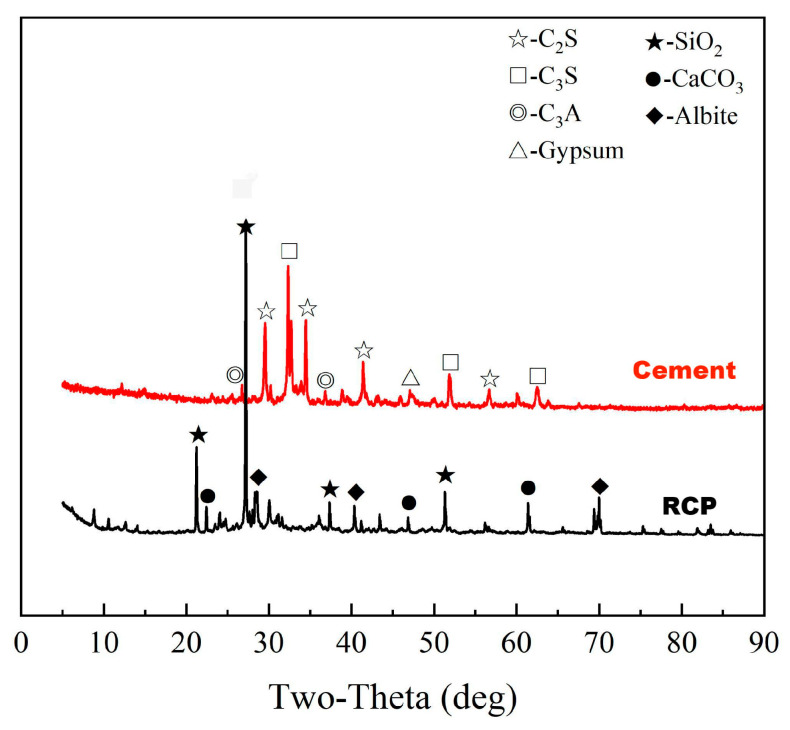
X-ray diffraction pattern of cement and RCP.

P.O42.5 ordinary silicate cement supplied by Jidong Cement Co., Ltd., (Datong, China) was used in the test. Table 1 and Table 2 show the main physical property indices and the main chemical composition of the cement, respectively. RCP was obtained from the discarded concrete blocks of the rehabilitated pavement, crushed by a jaw crusher, screened (through a 5 mm mesh screen), ball milled, and re-screened (through a 0.075 mm mesh screen), and the process of preparation is shown in Figure 1. The chemical composition of RCP was analyzed by X-ray fluorescence (XRF) analysis, and the results are shown in Table 2. It can be observed that the main chemical components of RCP include SiO_2_, CaO, and Al_2_O_3_. In addition, the mineral fraction of RCP was examined using an X-ray diffractometer (XRD) and the results are shown in Figure 2. The major mineral components identified in RCP include quartz, calcite, and feldspar. These components are capable of reacting with cement hydration products to form cementitious products. Therefore, RCP is potentially active.

#### 2.1.2. Fine Aggregate and Coarse Aggregate

In the experiment, ordinary river sand with a grain size ranging from 0.15 mm to 5 mm was used as the fine aggregate. The sand is properly graded and has a fineness modulus of 2.7. The coarse aggregate used was machine crushed stone with continuous grading from 4.75 mm to 26.0 mm. Detailed performance indices of the sand and coarse aggregate are shown in Table 3.

#### 2.1.3. Others

In order to increase the OH concentration of the slurry and to replenish the Ca^2+^ content, analytically pure Ca(OH)_2_ was used as a chemical exciter, specifically selected to stimulate the activity of the RCP (with potential to react with water and cement), produced by the Tianjin Beichen Fangzheng Reagent Factory (Tianjin, China). A high efficiency polycarboxylic acid water reducer was used to achieve a water reduction rate of approximately 30%.

### 2.2. Preparation of the Recycled Powder Concrete Samples

#### 2.2.1. Mixing Proportions

Based on the Technical Guidelines for Construction of Highway Cement Concrete Pavements (JTG/TF30-2014) [35], the original mix proportions were used to formulate the natural concrete (NC) according to the flexural strength of 5 MPa. The water–cement ratio was 0.37, the sand content was 34%, and the water-reducing agent dosage was 1.0%. In addition, the RCP was incorporated into the mix at replacement rates of 0%, 10%, 20%, and 30%, respectively, based on the original mix proportions. In addition, 2.5% Ca(OH)_2_ was selected as the activator, and the RCP was used to replace the cement at replacement rates of 10%, 20%, and 30%, respectively. The details of the mixes are listed in Table 4.

#### 2.2.2. Sample Preparation 

The procedure commenced with an initial blending of sand and coarse aggregate within a mixer for a duration of 2 min. Subsequently, cement and recycled micronized powder were introduced sequentially and mixed for 2 min to achieve homogeneity. Following this, a composite of water reducer, exciter, and test water was meticulously prepared and then introduced into the mixer for a 2-min cycle. Finally, the meticulously blended concrete was poured into molds with precision. The molded concrete specimens were then subjected to 2 min of controlled vibration on a vibrator, followed by the meticulous removal of excess mixture using a trowel, thereby ensuring strict adherence to the specified specimen dimensions.

### 2.3. Test Methods 

#### 2.3.1. Compressive Strength Test

In accordance with the Test Methods of Cement and Concrete for Highway Engineering (JTG 3420-2020) [36], the dimensions of the specimens were 100 mm × 100 mm × 100 mm. Each group consisted of three specimens that were cured to the specified test age. They were then removed from the curing room, surface water was wiped off, and the cubic compressive strength test was immediately conducted. During testing, the center of each specimen was aligned with the center of the lower compression plate of the testing machine. The load was applied uniformly and continuously at a controlled loading rate of 0.5 MPa/s.

#### 2.3.2. Flexural Tensile Strength Test

In accordance with the Test Methods of Cement and Concrete for Highway Engineering (JTG 3420-2020) [36], the specimens were sized 100 mm × 100 mm × 400 mm. After curing for the specified test age, each group of three specimens was removed from the curing chamber, surface moisture was wiped off, and immediate flexural tensile tests were performed. Two adjustable supports were arranged to ensure stability and uniformity of the contact surfaces, and the specimens were placed on the supports, laterally and geometrically aligned. The tests were performed with continuous and uniform loading at a controlled loading rate of 0.05 MPa/s.

#### 2.3.3. Low-Field NMR Test

The principle of the low-field NMR instrument is to convert the energy change signal of water molecules at resonance into the *T*_2_ relaxation value by the action of external gradient field, so that the water molecules in the magnetic field and the gradient field produce nuclear magnetic resonance phenomenon. According to the change of *T*_2_, a certain calculation and inversion are carried out to determine the pore structure characteristics in concrete.

The relaxation time *T*_2_ can be expressed by Equation (1) [37]:

(1) $$\frac{1}{T_{2}}=\frac{1}{T_{2B}}+\rho_{2}\frac{S}{V}+\frac{1}{T_{2D}}$$

In the formula: *T*_2*B*_—the volume relaxation time of the fluid and $\rho_{2}$—the transverse surface relaxation rate is a parameter to characterize the porous structure. The concrete material is generally selected as 0.001 cm/s, *S*—pore surface area, *V*—pore volume, and *T*_2*D*_—diffusion relaxation time of fluid.

In this experiment, saturated specimens are used, and *T*_2*B*_ and *T*_2*D*_ can be ignored in saturated structures [38]. Assuming the shape of the pore structure, the Equation (1) can be simplified as:

(2) $$\frac{1}{T_{2}}=\rho_{2}\frac{G}{r}$$

In the formula: *G*—pore shape factor, assuming that the pore structure is an ideal sphere, then *G* is 3 and *r*—is the aperture.

When $\rho_{2}$ and the shape of the pore structure are determined, the pore size *r* can be determined from the *T*_2_ value measured by the low-field NMR instrument (Suzhou Niumag Analytical Instrument Co., Ltd., Suzhou, China).

**Figure 3:**
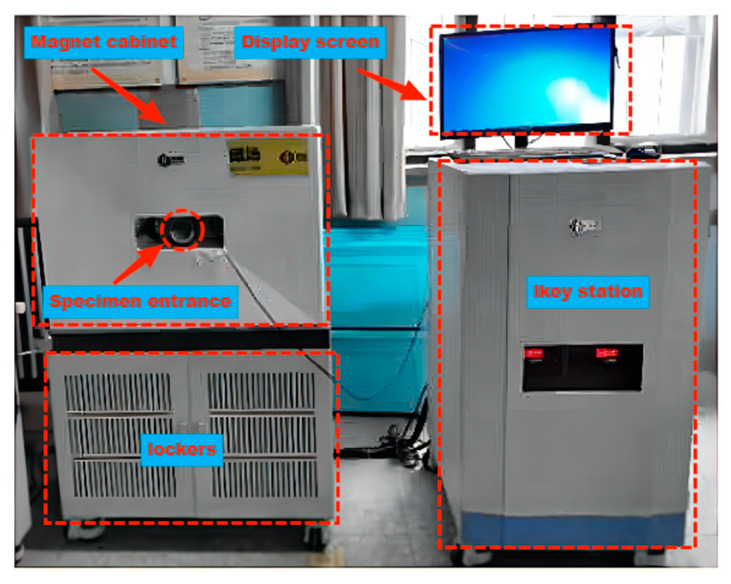
Low-field NMR instrument.

The specimen for this experiment has a cylindrical shape with a diameter and height of 50 mm. After reaching the specified curing age, the specimen is removed for vacuum saturation. The concrete specimen is first subjected to vacuum saturation for 8 h using a vacuum saturation device to ensure complete saturation. The specimens are then soaked in water for 24 h. The samples are then removed from the water saturation apparatus, wrapped in plastic wrap, and immediately placed in a pre-prepared water tank. Before the low-field NMR test is performed, the low-field NMR instrument is calibrated. The excess water on the surface of the plastic film is wiped off, and the samples are carefully positioned in the low-field NMR system for testing [39]. The low-field NMR test equipment is shown in Figure 3, and the parameter settings are shown in Table 5.

## 3. Results and Discussion

### 3.1. Mechanical Properties

**Figure 4:**
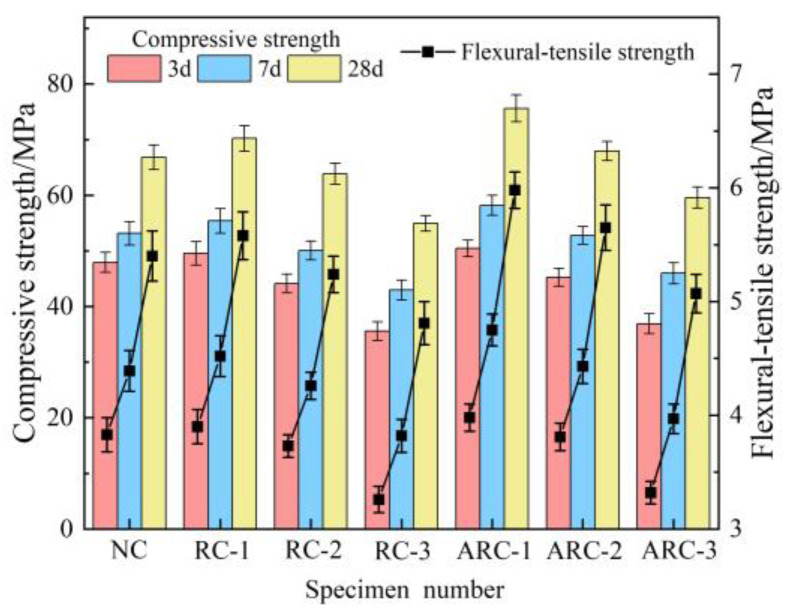
Mechanical properties of RCP concrete and ARC at various maintenance ages.

The mechanical properties of RCP concrete and ARC at different curing ages are shown in Figure 4. It can be observed from Figure 4 that with an increase in the amount of RCP, both RCP concrete and ARC show a trend of initially increasing and then decreasing compressive strength and flexural tensile strength at curing ages of 3 d, 7 d, and 28 d. The mechanical properties peak at a dosage of 10% RCP. Compared to NC, the compressive strength of RC-1 increased by 3.44%, 4.18%, and 5.07%, while the flexural tensile strength increased by 1.83%, 2.96%, and 3.33% at curing ages of 3 d, 7 d, and 28 d, respectively. Similarly, ARC-1 showed an increase in compressive strength of 5.26%, 9.44%, and 13.17% and an increase in flexural tensile strength of 3.92%, 8.20%, and 10.74% compared to NC. These results indicate that a reasonable amount of RCP (10%) contributes to the improvement of the mechanical properties of concrete. The promoting effect of RCP on the mechanical properties of concrete can be attributed to three factors. First, the active components SiO_2_ and Al_2_O_3_ in RCP react with Ca(OH)_2_ to form cementitious products such as calcium silicate hydrate (C-S-H) and calcium aluminate hydrate (C-A-H). These products effectively fill the internal pores of the concrete, improving its compactness and promoting strength development [40]. Second, deep ball milling increases the specific surface area of the RCP, allowing for improved dispersion and contact area with hydration. It also exposes unhydrated particles, thereby increasing the hydration activity of the RCP. Third, the presence of inert particles in the RCP has the potential to fill the small pores between the fine aggregates, thereby facilitating a micro-aggregate filling effect [41], which densifies the microstructure and consequently improves the mechanical properties of the concrete. When the amount of RCP exceeds 10%, there is a gradual decrease in the mechanical properties of the concrete. The lowest compressive and flexural tensile strengths of both RCP concrete and ARC were observed at each age when the amount of RCP was 30%. Specifically, compared to NC, RC-3 showed a reduction in compressive strength of 25.79%, 19.21%, and 17.76%, and a reduction in flexural tensile strength of 14.88%, 12.98%, and 10.93% at curing ages of 3 d, 7 d, and 28 d, respectively. In addition, ARC-1 showed a reduction in compressive strength of 23.02%, 13.51%, and 10.85% and a reduction in flexural tensile strength of 13.5%, 9.57%, and 6.11%, compared to NC. These results indicate that excessive incorporation of RCP (30%) significantly reduces the mechanical properties of the concrete. The detrimental effect of RCP on the mechanical properties of concrete can be attributed to two main factors. First, the RCP itself is loose, porous, and highly water-absorbent [42], resulting in the absorption of a significant amount of free water during mixing. As a result, the water availability for cement hydration is reduced, which to some extent inhibits the cement–hydration reaction. In addition, an excessive amount of RCP increases the number of interfacial transition zones within the concrete matrix, leading to a decrease in its mechanical properties [20]. Secondly, excessive incorporation of RCP leads to a reduction in the cement content of the concrete, and consequently the content of the active component of the volcanic ash is insufficient for a complete reaction with Ca(OH)_2_, resulting in a reduction in the formation of hydration products such as C-S-H. Furthermore, the excess amount of RCP fills concrete microcracks without undergoing secondary hydration reactions, thereby weakening the mechanical properties of the concrete.

It can also be seen from Figure 4 that the compressive and flexural tensile strengths of ARC were improved over RCP concrete for the same RCP dosage and age of maintenance. It is concluded that the inclusion of an appropriate amount of Ca(OH)_2_ is beneficial in stimulating the activity of RCP, thereby improving the mechanical properties of the concrete. The addition of Ca(OH)_2_ primarily increases the OH concentration in the slurry, which facilitates the formation of free unsaturated reactive bonds on the surface of RCP in an alkaline environment. As a result, the degree of polymerization of the SiO_2_ and Al_2_O_3_ network aggregates decreases, increasing their reactivity with the active components in the liquid phase of the system. Moreover, this process leads to an increased production of cementitious products, which ultimately stimulates the activity of the RCP and improves the mechanical properties of the concrete. Ca(OH)_2_, as an activator, introduces a significant amount of Ca^2+^ ions, replenishing the CaO content in the RCP and promoting the formation of silicate and other cementitious materials. Meanwhile, Ca(OH)_2_ can react with active SiO_2_, Al_2_O_3_ in a large amount of aluminum-silica glass in the RCP in a volcanic ash reaction to produce C-S-H and C-A-H with higher strength and water hardness.

**Figure 5:**
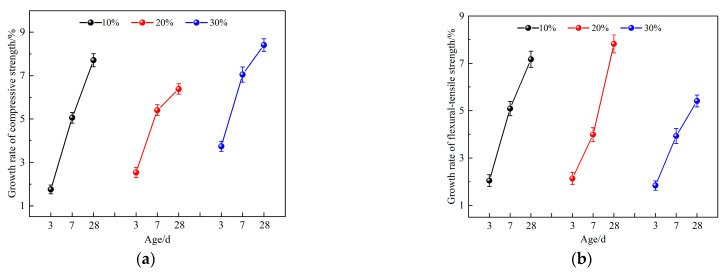
Growth rate of mechanical properties of ARC compared with RCP concrete. (**a**) compressive strength; (**b**) flexural tensile strength.

The growth rates of ARC over RCP concrete mechanical properties at the same RCP loading and curing age are shown in Figure 5. As shown in Figure 5, when the amount of RCP is 10%, the growth rates of compressive strength at the 3-day, 7-day and 28-day curing ages are 1.76%, 5.06% and 7.71%, respectively. Meanwhile, the corresponding growth rates of flexural tensile strength are 2.05%, 5.09%, and 7.17%, respectively. Similarly, when the amount of RCP is 20%, the growth rates of compressive strength at the same curing ages are 2.54%, 5.41%, and 6.39%, while the growth rates of flexural tensile strength are also 2.54%, 5.41%, and 6.39%, respectively. In addition, with 30% inclusion of RCP, the growth rates of compressive strength at the 3-day, 7-day, and 28-day curing ages are 3.74%, 7.05%, and 8.41%, while the growth rates of flexural tensile strength are 1.84%, 3.93%, and 5.41%, respectively. With the increase of curing age, the growth rate of compressive strength and flexural tensile strength increased, and the effect of alkali excitation on the mechanical properties of concrete became higher and higher. This is due to the fact that Ca(OH)_2_ itself has certain cementing properties, but its solubility is low, resulting in insignificant early strength growth of the concrete. With the prolongation of the curing time, a large amount of Ca^2+^ and the rest of the active substances in the system were dissolved, and a large amount of calcium aluminate and some gelatinous substances were formed, which led to the improvement of the late strength of the system [43].

### 3.2. Pore Structure Analysis

#### 3.2.1. Porosity

**Figure 6:**
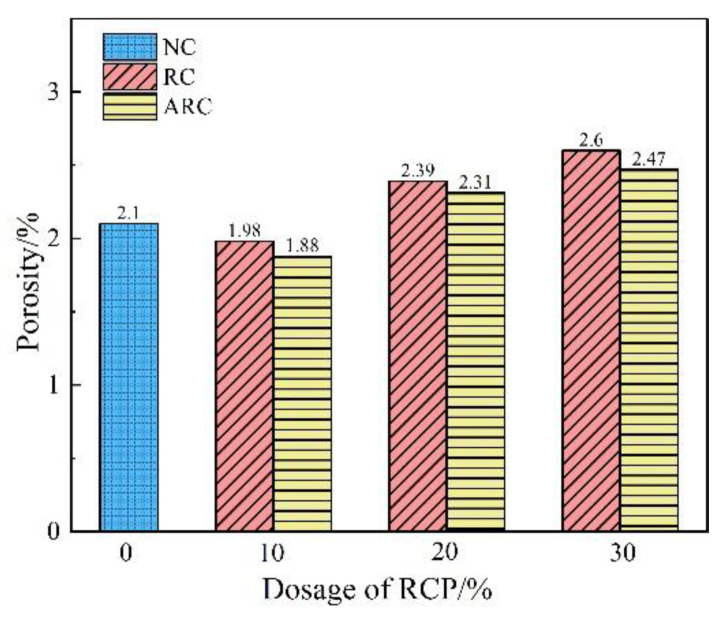
The variation of RCP concrete and ARC porosity with the content of RCP under the curing age of 28 d.

The variation patterns of RCP concrete and ARC porosity with RCP dosage at 28 d maintenance age are shown in Figure 6. According to Figure 6, the porosity of RCP concrete consistently exceeds that of ARC, regardless of RCP dosage. This difference is primarily due to the alkaline environment provided by ARC, which facilitates the increased hydration rate of C_2_S and C_3_S. The increased hydration results in the generation of a significant volume of C-S-H and C-A-H, which effectively fills the concrete pores and reduces the porosity of ARC [44]. Both RCP concrete and ARC show an initial decreasing trend in porosity with increasing RCP dosage, followed by an upward trend. At 10% RCP, RC-1 and ARC-1 reach the lowest porosity value, corresponding to a reduction of 5.71% and 10.48%, respectively, compared to NC. Subsequently, the porosity increases for both RCP concrete and ARC as the amount of RCP exceeds 10%. In particular, the porosity reaches its maximum at a dosage of 30%, with RC-3 and ARC-3 showing increases of 23.81% and 17.62%, respectively, compared to the NC specimens. On the one hand, the appropriate dosage of RCP (10%) effectively reduces the pore volume and increases the compactness of the concrete. This result is due to the secondary hydration reaction and micro-aggregate filling effect, in which the RCP occupies the tiny pores within the concrete [39]. On the other hand, excessive incorporation of RCP (30%) results in increased pore volume and decreased compactness. The reason for this result is that the road concrete itself has a low water–cement ratio and a high mixing consistency. In addition, the loose and porous nature of RCP, characterized by high water absorption, results in the absorption of a significant amount of free water during mixing [42]. In addition, it is difficult to remove internal air bubbles during vibration, which negatively affects the compactness of the concrete and ultimately increases its porosity.

**Figure 7:**
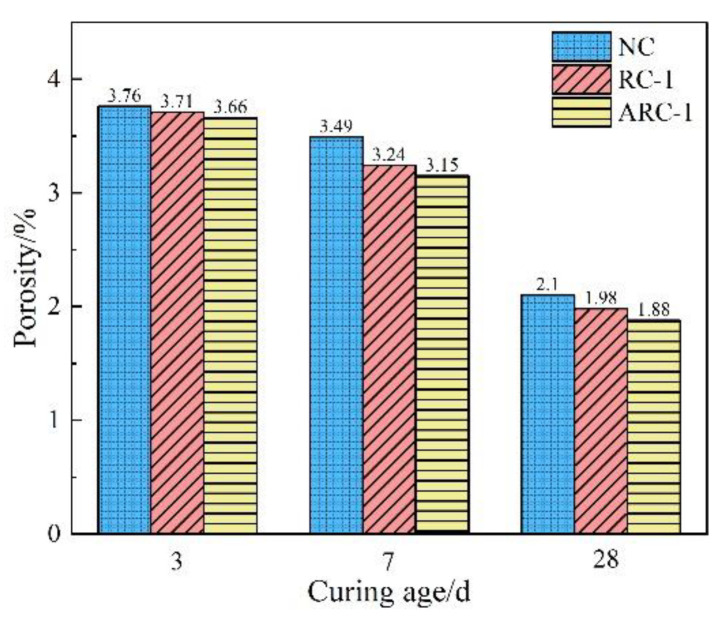
The variation of porosity of NC, RC-1, and ARC-1 with curing age.

The variation pattern of porosity in NC, RC-1, and ARC-1 with curing age are shown in Figure 7. It can be seen from Figure 7 that the porosity of concrete gradually decreases with increasing curing age. Furthermore, the porosity of ARC-1 shows a greater decrease compared to RC-1, indicating an increasing influence of alkali excitation on the porosity of concrete. The main reason for this phenomenon is that the alkali exciter, Ca(OH)_2_, is not readily soluble in water, which hinders its ability to dissolve rapidly during the early stages of the hydration reaction [43]. Over time, however, it can react with the cement and RCP in the active material. This reaction results in the formation of numerous hydration products such as C-S-H, C-A-H, AFt, etc. These products fill the internal pores of the concrete, making it denser and less porous. The porosity ranking, from highest to lowest, at each curing age is NC > RC-1 > ARC-1. This indicates that the incorporation of 10% RCP is beneficial for improving the compactness of concrete. Moreover, the compactness of concrete is further improved after alkali excitation.

#### 3.2.2. Pore Size Distribution

**Figure 8:**
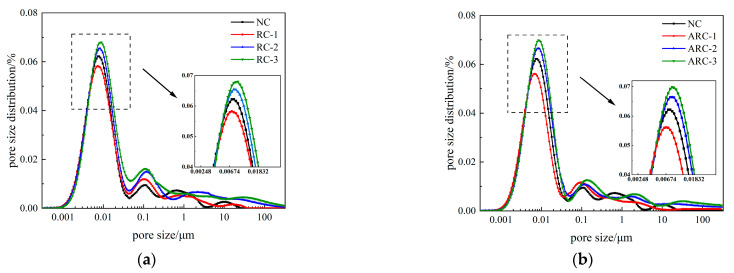
The pore size distribution curves of RCP concrete and ARC with different content of RCP. (**a**) RCP concrete; (**b**) ARC.

The pore size distribution curves of concrete containing different amounts of RCP at a curing age of 28 days are shown in Figure 8. It can be seen from Figure 8 that each RCP sample has at least two distinct peaks in the pore size distribution curves. The main peaks are distributed in the smaller pore sizes from 1 to 50 nm and the rest of the secondary peaks have very small peak areas and are distributed in the larger pore sizes. This means that the pores in the concrete samples are mainly dominated by small pore sizes, with few larger pore sizes. In Figure 8a, the main peak areas are in the order of RC-3, RC-2, NC, and RC-1, while in Figure 8b, the main peak areas are in the order of ARC-3, ARC-2, NC, and ARC-1. The most probable pore sizes corresponds to the peak pore size in the pore size distribution curve, which reflects the distribution of pore sizes. A larger most probable pore sizes indicates a higher number of large capillaries and greater internal pore connectivity [45]. With the addition of RCP ranging from 0% to 10%, the peak of the main peak decreases along with a reduction in pore volume. Specifically, in Figure 8a, the most available pore size of RCP concrete decreases from 7.838 nm to 7.312 nm, and in Figure 8b, the most available pore size of ARC decreases from 7.838 nm to 6.822 nm. These results suggest that a reasonable amount of RCP can effectively fill the internal pores of the concrete, thereby improving its mechanical properties. However, when the amount of RCP is further increased from 10% to 30%, the size of the main peak continues to increase, accompanied by an increase in pore volume. The most available pore size of RCP concrete in Figure 8a increases from 7.312 nm to 9.006 nm, and the most available pore size of ARC in Figure 8b increases from 6.822 nm to 8.402 nm. This indicates that the excessive addition of RCP leads to an increase in the internal pores of the concrete and a subsequent decrease in its mechanical properties.

**Figure 9:**
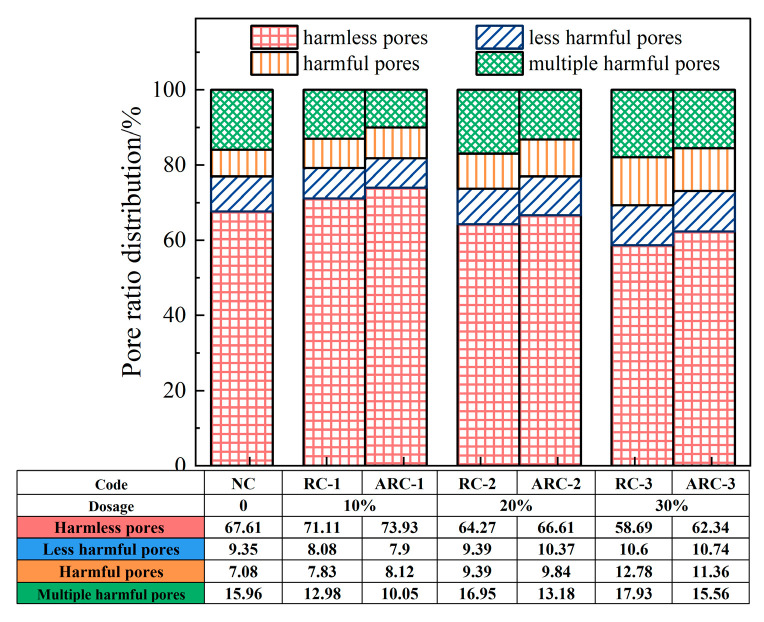
Pore size distribution of RC and ARC with different RCP content.

According to the classification of pore classes in concrete by Wu [46], different pore sizes have different effects on concrete performance, categorized as harmless pores (<20 nm), less harmful pores (20~50 nm), harmful pores (50~200 nm), and multiple harmful pores (>200 nm). The distribution pattern of different types of pore size percentages of concrete with different dosages of RCP is shown in Figure 9. According to Figure 9, It can be observed that the percentage of harmless pores first increases and then decreases, while the percentage of multiple harmful pores first decreases and then increases with increasing RCP content. For RCP proportions ranging from 0% to 10%, the growth rates of the harmless pores for RC-1 and ARC-1 are 5.18% and 9.35%, respectively, while the reduction rates of the multiple harmful pores are 18.67% and 37.05%, respectively. For RCP proportions from 10% to 30%, the reduction rates of the harmless pores for RC-3 and ARC-3 are 17.47% and 15.68%, respectively. Meanwhile, the growth rates of the multiple harmful pores are 38.14% and 54.83%, respectively. These results suggest that the incorporation of a reasonable amount of RCP (10%) effectively improves the distribution of pore classes in concrete. However, excessive incorporation of RCP (30%) adversely affects the distribution of pore classes in concrete. Under RCP contents of 10%, 20%, and 30%, the growth rates of the harmless pores for ARC compared to RCP concrete are 3.97%, 3.64%, and 6.22%, respectively, while the reduction rates of the multiple harmful pores are 22.57%, 22.24%, and 13.22%, respectively. This indicates that under the influence of Ca(OH)_2_, the excitation of the secondary volcanic ash reaction of RCP leads to the formation of C-S-H gel. This reaction not only increases the strength of the concrete, but also effectively fills the internal pores, leading to an increase in the proportion of harmless pores and a decrease in the proportion of multiple harmful pores. As a result, the pore size distribution is significantly improved.

**Figure 10:**
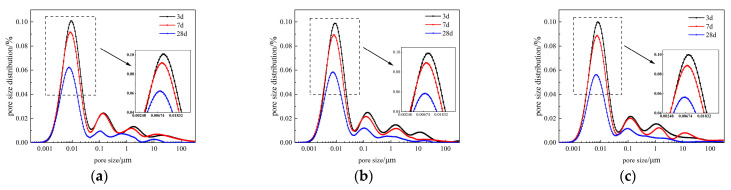
Pore size distribution curves of NC, RC-1, and ARC-1 at different curing ages. (**a**) NC; (**b**) RC-1; (**c**) ARC-1.

The pore size distribution curves of NC, RC-1, and ARC-1 at different curing ages are shown in Figure 10. From Figure 10, it can be observed that as the curing age increases, both the peak area of the main peak and the secondary peak gradually decrease, and the secondary peak becomes smoother. As shown in Figure 10a, the most probable pore sizes of NC at curing ages of 3 days, 7 days, and 28 days are 9.653 nm, 8.402 nm, and 7.838 nm, respectively. Similarly, as shown in Figure 10b, the most probable pore sizes of RC-1 at the same curing ages are 9.006 nm, 8.402 nm, and 7.312 nm, respectively. In addition, Figure 10c shows that for ARC-1, the most probable pore sizes at the same curing ages are 7.838 nm, 7.312 nm, and 6.822 nm, respectively. As the curing age increases, the peak values and most probable pore sizes of the main peak gradually decrease, indicating a reduction in pore volume. This means that the pore size of the concrete continues to refine as the curing age increases. This process reflects the filling of the pores by the gel formed due to reactions within the pore during the hydration process, thereby contributing to a denser concrete structure [14].

**Figure 11:**
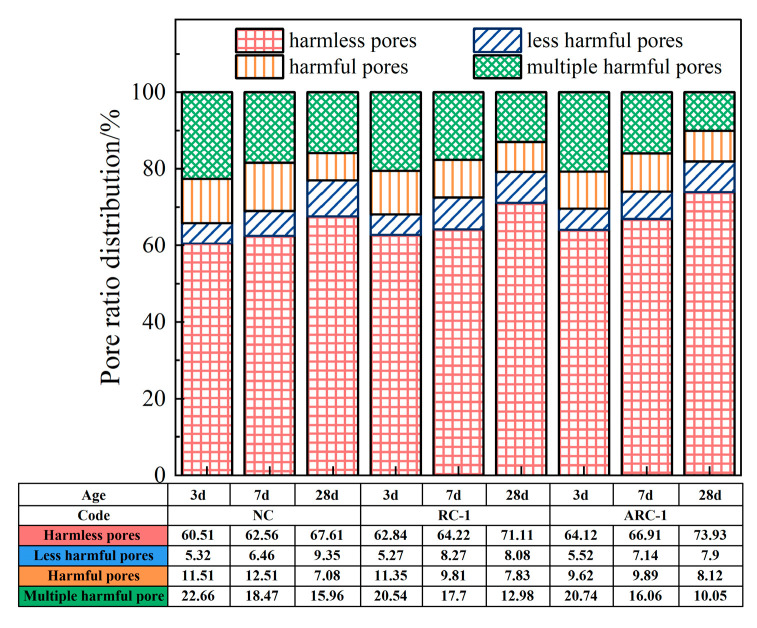
Pore size distribution of NC, RC-1, and ARC-1 at different curing ages.

The pore size distribution trends of NC, RC-1, and ARC-1 at each curing age are shown in Figure 11. As shown in Figure 11, it is observed that the proportion of harmless pores in NC, RC-1, and ARC-1 gradually increases, while the proportion of multiple harmful pores gradually decreases with increasing curing age. At the curing ages of 3 days, 7 days, and 28 days, the percentage of harmless pores in NC is recorded as 60.51%, 62.56%, and 67.61%, respectively, while the percentage of multiple harmful pores is measured as 22.66%, 18.47%, and 15.96%, respectively. Similarly, for RC-1, the percentage of harmless pores is found to be 62.84%, 64.22%, and 71.11%, and the percentage of multiple harmful pores is found to be 20.54%, 17.7%, and 12.98%, respectively. In the case of ARC-1, the percentages of harmless pores are 64.12%, 66.91%, and 73.93%, and the percentages of multiple harmful pores are 20.74%, 16.06%, and 10.05%, respectively. These results indicate that as the age increases, the degree of hydration increases and more hydration products are generated and continue to fill the capillary pores, resulting in a decrease in the number of capillary pores and a denser material. Therefore, as the age increases, the skeletal density increases, the pore space decreases, and the pore size decreases.

### 3.3. Gray Correlation Entropy Analysis

The relationship between the mechanical properties of RCP and fine-scale pore structure characteristics was investigated using gray correlation entropy analysis [47,48,49]. The purpose of this analysis was to identify the primary pore structure factors that influence the mechanical properties. The gray correlation entropy analysis is a method to measure the degree of correlation between factors based on the similarity or dissimilarity of the development trend between the factors, without having to take into account the specific distribution of the data, and the calculation is convenient and reliable. The calculation steps for the gray correlation entropy analysis are outlined below:

1. Determine the order of fundamental parameters:

The mechanical properties of recycled powder concrete, denoted as $Y_{i}=\{Y_{i}(k)|\;k=1,2,\cdots ,n\},\;i=1,2,\cdots ,m$, are represented in the reference sequence data. The pore structure factors influencing the mechanical properties in the above reference sequence, denoted as $X_{j}=\{X_{j}(k)|\;k=1,2,\cdots ,n\},\;j=1,2,\cdots ,l$, are represented in the comparison sequence data. Where *n* = 7, *m* = 2, and *l* = 5.

2. Dimensionless processing of data in each series:
  (3) $$X_{j}^{\prime }=\frac{X_{j}(k)}{\bar{X_{j}}}(k=1,2,\cdots ,n;\;j=1,2,\cdots ,l)$$
  (4) $$Y_{i}^{\prime }=\frac{Y_{i}(k)}{\bar{Y_{i}}}(k=1,2,\cdots ,n;\;i=1,2,\cdots ,m)$$
3. Calculate the absolute difference between the reference sequence and the comparison sequence.
  (5) $$\Delta_{ij}(k)=\left|Y_{i}(k)-X_{j}(k)\right|=(\Delta_{ij}(1),\Delta_{ij}(2),\cdots ,\Delta_{ij}(n))$$
4. Determine the maximum and minimum values of the absolute difference, denoted as:
  (6) $$M=\underset{i}{\max}\;\underset{j}{\max}\;\Delta_{ij}(k),m=\underset{i}{\min}\;\underset{j}{\min}\;\Delta_{ij}(k)$$
5. Computing the number of correlation coefficients.
  (7) $$\mu_{ij}(k)=\frac{m+\xi M}{\Delta_{ij}(k)+\xi M}$$
6. Calculate the gray relation entropy.
  (8) $$p_{ij}(k)=\frac{\mu_{ij}(k)}{\sum_{k=1}^{n}\mu_{ij}(k)}$$
  (9) $$S_{ij}(k)=-\sum_{k=1}^{n}P_{ij}(k)\ln P_{ij}(k)$$
7. Calculate the gray entropy correlation grade.
  (10) $$E_{ij}(k)=\frac{S_{ij}(k)}{S_{\max}}$$
  where: $S_{\max}$—maximum entropy of difference information column, $S_{\max}=\ln n$.

The data of NC, RC-1, RC-2, RC-3, ARC-1, ARC-2, and ARC-3 specimens at the age of 28 d of maintenance were used as the basis for the theoretical calculation of the gray entropy correlation. Compressive strength and flexural tensile strength were selected as the reference sequence *Y_i_*, and porosity, harmless pores, less harmful pores, multiple harmful pores, and harmful pores were selected as the comparison sequence *X_j_*. The parameter sequences are shown in Table 6.

Based on Table 6, the gray relation entropy and gray entropy correlation grade of pore characteristic parameters on the mechanical properties of concrete mixed with recycled micronized concrete were calculated by applying the gray correlation entropy analysis method as shown in Equations (3)–(10), and the results are shown in Table 7.

From Table 7, it can be seen that the compressive strength and flexural tensile strength of RCP concrete are in the same order of gray entropy correlation grade for each pore characteristic parameter of low-field NMR, i.e., harmless pore > porosity > multiple harmful pores > less harmful pore > harmful pore. According to the theory of gray correlation entropy analysis, the greater the value of gray entropy correlation grade, the greater the influence of this influence factor on the reference column. It can be seen that the harmless pores have the greatest influence on the mechanical properties of concrete with RCP.

### 3.4. GM (1, N) Model

The gray prediction model is generated by accumulating the original series once so that the discrete data have a certain regularity, and the differential equation for the newly generated series is the GM prediction model, which is calculated using the least squares method to obtain the relevant parameters of the model [33]. The GM (1, N) model is more commonly used among the gray prediction models, which is used to describe multivariate systems that respond to (N − 1) influences on the first-order derivatives of the main variables. The specific computational steps of the GM (1, N) prediction model are as follows:

8. Determine the system characteristic data sequence A and the sequence of related factors as:

(11) $$\left\{\left.\begin{array}{l} X_{2}{}^{\left(0\right)}=\left(x_{2}{}^{\left(0\right)}(1),x_{2}{}^{\left(0\right)}(2),\cdots x_{2}{}^{\left(0\right)}(n)\right)\\ X_{3}{}^{\left(0\right)}=\left(x_{3}{}^{\left(0\right)}(1),x_{3}{}^{\left(0\right)}(2),\cdots x_{3}{}^{\left(0\right)}(n)\right)\\ \cdots \\ X_{N}{}^{\left(0\right)}=\left(x_{N}{}^{\left(0\right)}(1),x_{N}{}^{\left(0\right)}(2),\cdots x_{N}{}^{\left(0\right)}(n)\right) \end{array}\right\}\right.$$

9. Find its corresponding once accumulated generating sequence (AGO).
  (12) $$X_{i}{}^{\left(1\right)}=\left(x_{i}{}^{\left(1\right)}(1),x_{i}{}^{\left(1\right)}(2),\cdots x_{i}{}^{\left(1\right)}(n)\right)\;i=1,2\ldots ,N$$
  where: $x_{i}{}^{\left(1\right)}(n)=\sum_{j=1}^{n}x_{i}{}^{\left(0\right)}(j)$

10. *A* is the immediate neighbor of *B* to generate a sequence of mean values.
  (13) $$Z_{1}{}^{\left(1\right)}=\left(z_{1}{}^{\left(1\right)}(2),z_{1}{}^{\left(1\right)}(3),\cdots z_{1}{}^{\left(1\right)}(n)\right)$$
  where $z_{1}{}^{\left(1\right)}(n)=\frac{1}{2}\left[x_{1}{}^{\left(1\right)}(n)+x_{1}{}^{\left(1\right)}(n-1)\right]$

11. Establish the GM (1, N) model.
  (14) $$x_{1}{}^{\left(0\right)}(k)+az_{1}{}^{\left(1\right)}(k)=\sum_{i=2}^{n}b_{i}x_{i}{}^{\left(1\right)}(k)$$
  where a is the development coefficient, $b_{i}x_{i}{}^{\left(1\right)}(k)$ is the driving term, $b_{i}$ is the driving coefficient, and $\overset{\wedge }{a}={\left[a,b_{1},b_{2}\cdots b_{n}\right]}^{T}$ is the parameter column that can be obtained by least squares $\overset{\wedge }{a}={\left(B^{T}B\right)}^{-1}B^{T}Y$, where:
  (15) $$B=\left[\begin{array}{llll} -z_{1}{}^{\left(1\right)}(2) & x_{2}{}^{\left(1\right)}(2) & \cdots & x_{n}{}^{\left(1\right)}(2)\\ -z_{1}{}^{\left(1\right)}(3) & x_{2}{}^{\left(1\right)}(3) & \cdots & x_{n}{}^{\left(1\right)}(3)\\ \vdots & \vdots & & \vdots \\ -z_{1}{}^{\left(1\right)}(n) & x_{2}{}^{\left(1\right)}(n) & \cdots & x_{n}{}^{\left(1\right)}(n)\; \end{array}\right]\;\;Y=\left[\begin{array}{l} x_{1}{}^{\left(0\right)}(2)\\ x_{1}{}^{\left(0\right)}(3)\\ \vdots \\ x_{1}{}^{\left(0\right)}(n) \end{array}\right]$$

Through the gray relational entropy analysis, the functional relationship between the system characteristic data sequence and the related factor sequence is established. The data of the top three pore characteristic parameters (harmless pore, porosity, and multiple harmful pores) and mechanical properties (compressive strength and flexural strength) of RCP concrete are used as the modeling set, and the data are substituted into the GM (1, 4) gray prediction model. The compressive strength prediction model of RCP concrete is obtained by the Equations (11)–(15):

(16) $${\overset{\wedge }{x}}_{1}{}^{\left(0\right)}(k)=-2.295Z_{1}{}^{\left(1\right)}(k)+1.147x_{2}{}^{\left(1\right)}(k)-1.636x_{3}{}^{\left(1\right)}(k)-0.444x_{4}{}^{\left(1\right)}(k)$$

Flexural tensile strength prediction model:

(17) $${\overset{\wedge }{x}}_{1}{}^{\left(0\right)}(k)=-2.249Z_{1}{}^{\left(1\right)}(k)+0.077x_{2}{}^{\left(1\right)}(k)+0.179x_{3}{}^{\left(1\right)}(k)-0.006x_{4}{}^{\left(1\right)}(k)$$

After the GM (1, 4) gray prediction model was established, the reasonableness of the model simulation and prediction performance was evaluated using accuracy tests such as the mean relative error and the posterior difference ratio [50]. The criteria for evaluating the accuracy of the gray model are shown in Table 8.

The error test of the GM (1, 4) prediction model of compressive strength and flexural tensile strength of RCP concrete is Table 9 and Table 10, respectively. From Table 8, Table 9 and Table 10, it can be seen that the average relative error of compressive strength prediction of RCP concrete is 1.519%, which meets the requirement of relative error of the multivariate gray prediction model that is less than 15%, and the accuracy reaches level 2. The posterior error ratio is 0.189, and the accuracy reaches the first level. The average relative error of flexural tensile strength prediction of RCP concrete is 1.423%, and the accuracy reaches the second level. The posterior error ratio is 0.255, and the accuracy reaches the first level. Therefore, it can be seen that the model is a prediction model with better accuracy, and each index meets the accuracy requirements of the model. In order to improve the validity and robustness of the model, this paper considers using the data and samples of RCP concrete with curing ages of 3 d and 7 d for validation. Table 11 shows the raw data of pore characteristic parameters and strength of RCP concrete at 3 d and 7 d curing ages. The data in Table 11 were substituted into the compressive strength and flexural tensile strength prediction models of the RCP concrete, and the prediction results are shown in Table 12 and Table 13. It can be seen from Table 12 and Table 13 that the average relative errors of the compressive strength prediction of the RCP concrete at the curing ages of 3 d and 7 d are 3.082% and 1.706%, respectively, and the posterior difference ratios are 0.119 and 0.171. It has a flexural tensile strength prediction average relative error of 2.336% and 1.696%, respectively, and the a posteriori difference ratio value is 0.166 and 0.148, respectively. The average relative error precision of compressive strength and flexural tensile strength of RCP concrete are both level 2, and the precision of the a posteriori difference ratio are all level 1. As a result, it is verified that the GM (1, 4) prediction model has high accuracy, and the model can be applied to the prediction of mechanical properties of RCP concrete and related experiments for reference.

## 4. Conclusions

In this paper, the GM (1, 4) prediction model is established by studying the compressive strength and flexural tensile strength of RCP concrete and analyzing the pore structure characteristics by low-field NMR. The main conclusions are as follows:

1. With the increase of RCP, the mechanical properties of concrete first increased and then decreased. When a reasonable amount of RCP (10%) is added, the secondary hydration reaction and micro-aggregate filling effect of RCP are beneficial to reduce the porosity of concrete and improve the mechanical properties of concrete. Excessive incorporation of RCP (30%) will cause the porosity of concrete to increase and the compactness to decrease, thereby reducing the mechanical properties of concrete.
2. The incorporation of 2.5% Ca(OH)_2_ is beneficial to stimulate the activity of RCP, improve the pore size distribution of concrete, reduce the porosity, and improve the compactness, thus improving the mechanical properties of ARC. As the curing age increases, the RCP is fully hydrated, the pore size in the concrete is further refined, the structure becomes denser, and the mechanical properties are improved. At the same time, the effect of alkali excitation on the mechanical properties of concrete becomes higher and higher.
3. The compressive strength and flexural tensile strength of RCP concrete are in the same order of gray entropy correlation degree of each pore characteristic parameter of low-field NMR, that is, harmless pore > porosity > multiple harmful pores > less harmful pore > harmful pore.
4. The average relative errors of the GM (1, 4) model for predicting the compressive strength and flexural strength of RCP concrete are 1.519% and 1.423%, respectively, and the accuracy is level 2. The posterior error ratios are 0.189 and 0.255, respectively, and the accuracy is one level. The established predictive GM (1, 4) model can accurately predict the mechanical properties of RCP concrete.

## Figures and Tables

**Table 1:** Physical properties index of cement.

Strength Grade	Apparent Density (g/cm^3^)	Specific Surface Area (m^2^/kg)	Setting Time (min)		Compressive Strength (MPa)		Flexural Strength (MPa)	
			Initial Setting	Final Setting	3 d	28 d	3 d	28 d
42.5	3.03	360	95	265	24.8	45.97	4.4	8.07

**Table 2:** Main chemical composition of cement and RCP (%).

	SiO_2_	CaO	Al_2_O_3_	Fe_2_O_3_	MgO	SO_3_	Ignition Loss
Cement	23.44	55.01	7.19	2.96	2.24	2.87	2.86
RCP	50.6	19.9	14.2	5.9	3.16	1.03	-

**Table 3:** Basic performance index of natural river sand and coarse aggregate.

	Apparent Density (kg/m^3^)	Stacking Density (kg/m^3^)	Void Ratio (%)	Mud Content (%)	Crushing Value (%)	Fineness Modulus
Fine Aggregate	2640	1510	41	2.3	-	2.7
Coarse Aggregate	2860	1450	45	0.8	8	-

**Table 4:** Mix proportions of recycled powder concrete (kg/m^3^).

No.	Water-Cement Ratio	RCP	Cement	Water	Sand	Coarse Aggregate	Water Reducer	Ca(OH)_2_
NC	0.37	-	319	118	667	1295	3.19	-
RC-1	0.37	31.9	287.1	118	667	1295	3.19	-
RC-2	0.37	63.8	255.2	118	667	1295	3.19	-
RC-3	0.37	95.7	223.3	118	667	1295	3.19	-
ARC-1	0.37	31.9	287.1	118	667	1295	3.19	0.798
ARC-2	0.37	63.8	255.2	118	667	1295	3.19	1.595
ARC-3	0.37	95.7	223.3	118	667	1295	3.19	2.393

**Table 5:** Low-field NMR equipment parameter setting.

Magnetic Field Intensity/T	Master Frequency/MHz	Scanned Area/mm	TE /ms	TW /ms	NS	NECH	PRG
0.5 ± 0.08	21.3	0~60	0.35	1000	4	13,000	3

**Table 6:** The specific parameter sequence of each concrete mix ratio.

Specimen Number	Comparison Sequence/%					Reference Sequence/MPa	
	Porosity	Harmless Pores	Less Harmful Pores	Harmful Pores	Multiple Harmful Pores	Compressive Strength	Flexural Tensile Strength
NC	2.1	67.61	9.35	7.08	15.96	66.82	5.4
RC-1	1.98	71.11	8.08	7.83	12.98	70.21	5.58
RC-2	2.39	64.27	9.39	9.39	16.95	63.86	5.24
RC-3	2.6	58.69	10.6	12.78	17.93	54.95	4.81
ARC-1	1.88	73.93	7.9	8.12	10.05	75.62	5.98
ARC-2	2.31	66.61	10.37	9.84	13.18	67.94	5.65
ARC-3	2.47	62.34	10.74	11.36	15.56	59.57	5.07

Note: The two sub-columns of the reference series are calculated by selecting only one item to be examined with all the items of the comparison series.

**Table 7:** Gray relation entropy and gray entropy correlation grade of pore characteristic parameters on mechanical properties of concrete.

Pore Characteristic Parameters	Compressive Strength		Flexural Tensile Strength	
	Gray Relation Entropy	Gray Entropy Correlation Grade	Gray Relation Entropy	Gray Entropy Correlation Grade
porosity	1.9396	0.9968	1.9399	0.9969
harmless pores	1.9438	0.9989	1.9439	0.9990
less harmful pores	1.9030	0.9779	1.9046	0.9788
harmful pores	1.8709	0.9615	1.8770	0.9646
multiple harmful pores	1.9153	0.9843	1.9199	0.9866

**Table 8:** Gray model accuracy judgment standard.

Accuracy Class	Mean Relative Deviation	Posterior Difference Ratio
first	0.01	0.35
second	0.05	0.5
third	0.1	0.65
fourth	0.2	0.8

**Table 9:** Compressive strength of RCP concrete at 28 d of curing age GM (1, 4) prediction model error test.

Sequence Number	Experimental Value	Predicted Value	Residual Error	Relative Error	Mean Relative Deviation	Posterior Difference Ratio
1	66.82	66.82	0	0	1.519%	0.189
2	70.21	72.546	2.336	3.496%		
3	63.86	63.105	−0.755	−1.076%		
4	54.95	55.437	0.487	0.762%		
5	75.62	76.540	0.92	1.675%		
6	67.94	66.283	−1.657	−2.191%		
7	59.57	60.543	0.973	1.432%		

**Table 10:** Flexural tensile strength of RCP concrete at 28 d of curing age GM (1, 4) prediction model error test.

Sequence Number	Experimental Value	Predicted Value	Residual Error	Relative Error	Mean Relative Deviation	Posterior Difference Ratio
1	5.4	5.4	0	0	1.423%	0.255
2	5.58	5.757	0.177	3.274%		
3	5.24	5.186	−0.054	−0.959%		
4	4.81	4.842	0.032	0.602%		
5	5.98	6.052	0.072	1.494%		
6	5.65	5.520	−0.130	−2.178%		
7	5.07	5.152	0.082	1.455%		

**Table 11:** Pore characteristic parameters and strength of RCP concrete at curing ages of 3 d and 7 d.

Specimen Number	Porosity/%		Harmless Pores/%		Multiple Harmful Pores/%		Compressive Strength/MPa		Flexural Tensile Strength/MPa	
	3 d	7 d	3 d	7 d	3 d	7 d	3 d	7 d	3 d	7 d
NC	3.76	3.49	60.51	62.56	22.66	18.47	47.92	53.16	3.83	4.39
RC-1	3.71	3.24	62.84	64.22	20.54	17.7	49.57	55.38	3.9	4.52
RC-2	3.88	3.68	57.27	60.27	23.95	19.95	44.13	50.06	3.73	4.26
RC-3	4.12	3.99	50.69	58.69	27.93	23.93	35.56	42.95	3.26	3.82
ARC-1	3.66	3.15	64.12	66.91	20.74	16.06	50.44	58.18	3.98	4.75
ARC-2	3.8	3.47	59.61	63.61	22.18	18.18	45.25	52.77	3.81	4.43
ARC-3	4.06	3.76	53.34	60.34	25.56	21.56	36.89	45.98	3.32	3.97

**Table 12:** Error test of GM (1, 4) prediction model for compressive strength of RCP concrete at curing ages of 3 d and 7 d.

Sequence Number	Experimental Value		Predicted Value		Mean Relative Deviation		Posterior Difference Ratio	
	3 d	7 d	3 d	7 d	3 d	7 d	3 d	7 d
1	47.92	53.16	47.92	53.16	3.082%	1.706%	0.119	0.171
2	49.57	55.38	48.12	52.6				
3	44.13	50.06	42.94	49.29				
4	35.56	42.95	37.12	43.11				
5	50.44	58.18	52.83	57.28				
6	45.25	52.77	43.79	53.52				
7	36.89	45.98	38.22	46.92				

**Table 13:** Error test of GM (1, 4) prediction model for flexural tensile strength of RCP concrete at curing ages of 3 d and 7 d.

Sequence Number	Experimental Value		Predicted Value		Mean Relative Deviation		Posterior Difference Ratio	
	3 d	7 d	3 d	7 d	3 d	7 d	3 d	7 d
1	3.83	4.39	3.83	4.39	2.336%	1.696%	0.166	0.148
2	3.9	4.52	3.99	4.37				
3	3.73	4.26	3.64	4.21				
4	3.26	3.82	3.35	3.76				
5	3.98	4.75	4.14	4.67				
6	3.81	4.43	3.74	4.49				
7	3.32	3.97	3.42	4.08				

## Data Availability

The general data are included in the article. Additional data are available on request.
